# Robert Fulton Clokey in Relation to the Irish Poor Law

**Published:** 1869-02

**Authors:** 


We are in receipt of a pamphlet of 24 pages written by our friend Robert Fulton Clokey, of Dublin, in relation to Public Health, Medical Relief and Sanitary Arrangements, under the ***Irish Poor Law*** and ***Medical Charities***; also a report from the

Dublin Quarterly Journal of Medical Science. We have been interested in our perusal of this pamphlet and gratified at the benevolence and liberality that the laws of Ireland bestow upon the sick and destitute of all classes, using the language of the writer: "Ireland is at present in the enjoyment of a system of medical relief equal, if not superior, to any country in Europe, and certainly very much in advance of that which prevails in England; that the organization of the system, which is comparatively inexpensive, is equally applicable to the most crowded city and the wildest and most remote country or mountain district; that adequate medical relief is accessible to all poor persons needing it, without delay or difficulty," etc., etc. The article is able written, and we regret the want of room for a more lengthy notice.

